# Bioactive Secondary Metabolites from the Red Sea Marine Verongid Sponge *Suberea* Species

**DOI:** 10.3390/md13041621

**Published:** 2015-03-24

**Authors:** Lamiaa A. Shaala, Diaa T. A. Youssef, Jihan M. Badr, Mansour Sulaiman, Alaa Khedr

**Affiliations:** 1 Natural Products Unit, King Fahd Medical Research Center, King Abdulaziz University, Jeddah 21589, Saudi Arabia; E-Mail: lshalla@kau.edu.sa; 2 Suez Canal University Hospital, Suez Canal University, Ismailia 41522, Egypt; 3 Department of Natural Products, Faculty of Pharmacy, King Abdulaziz University, Jeddah 21589, Saudi Arabia; E-Mail: jibrahim@kau.edu.sa; 4 Department of Pharmacognosy, Faculty of Pharmacy, Suez Canal University, Ismailia 41522, Egypt; 5 Department of Pharmacology, Faculty of Medicine, King Abdulaziz University, Jeddah 21589, Saudi Arabia; E-Mail: misulaiman@kau.edu.sa; 6 Department of Pharmaceutical Chemistry, Faculty of Pharmacy, King Abdulaziz University, Jeddah 21589, Saudi Arabia; E-Mail: khedr1961@gmail.com

**Keywords:** Red Sea Verongid sponge, *Suberea* species, dibrominated alkaloids, subereamollines C and D, antimigratory and antiproliferative activities, breast cancer cell line, HeLa cell line

## Abstract

In a continuation of our efforts to identify bioactive compounds from Red Sea Verongid sponges, the organic extract of the sponge *Suberea* species afforded seven compounds including two new dibrominated alkaloids, subereamollines C and D (**1** and **2**), together with the known compounds aerothionin (**3**), homoaerothionin (**4**), aeroplysinin-1 (**5**), aeroplysinin-2 (**6**) and a revised subereaphenol C (**7**) as ethyl 2-(2,4-dibromo-3,6-dihydroxyphenyl)acetate. The structures of the isolated compounds were assigned by different spectral data including optical rotations, 1D (^1^H and ^13^C) and 2D (COSY, multiplicity-edited HSQC, and HMBC) NMR and high-resolution mass spectroscopy. Aerothionin (**3**) and subereaphenol C (**7**) displayed potent cytotoxic activity against HeLa cell line with IC_50_ values of 29 and 13.3 µM, respectively. In addition, aeroplysinin-2 (**6**) showed potent antimigratory activity against the human breast cancer cell line MDA-MB-231 with IC_50_ of 18 µM. Subereamollines C and D are new congeners of the previously reported compounds subereamollines A and B with methyl ester functionalities on the side chain. These findings provide further insight into the biosynthetic capabilities of members of the genus *Suberea* and the chemical diversity as well as the biological activity of these compounds.

## 1. Introduction

**Figure 1:**
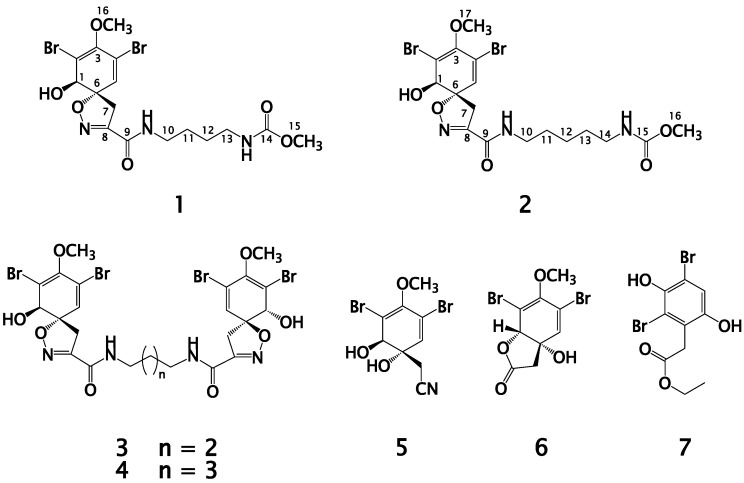
Structures of compounds **1**–**7**.

The phylum Porifera (sponges) has been considered as a gold mine for the chemists and has been considered as the most prolific source of secondary metabolites [1]. More novel bioactive compounds are obtained from members of this phylum each year than from any other marine taxon. These compounds showed diverse array of biological activities [1]. Bromotyrosine alkaloids are commonly encountered in marine sponges of the order Verongida [2,3,4,5,6,7,8,9,10]. These compounds displayed different biological activities including antifungal [2], antibacterial [3,4,5], cytotoxic [6,7,8,9] and enzyme inhibitory effects [10]. Our previous work on members of the Red Sea Verongid sponges led to the identification of different bioactive secondary metabolites [11,12,13,14,15,16]. As a continuation of our ongoing effort aimed to identify biologically active secondary metabolites from the marine Red Sea Verongid sponges [11,12,13,14,15,16], the alcoholic extract of the Red Sea sponge *Suberea* species was investigated. Members of the genus *Suberea* (order Verongida, family Aplysinellidae) are well known for their dibromotyrosine-derived secondary metabolites, halogenated compounds, polyaromatic alkaloids as well as terpenoidal compounds [1,9,11,12,13,14,15,17,18,19,20,21,22]. Several biological activities for these compounds were reported including antimicrobial, cytotoxic, enzyme inhibitory and anticancer effects [1,9,11,12,13,14,15,17,18,19,20,21,22]. In this work, we report the isolation of two new alkaloids, subereamollines C (**1**) and D (**2**) from the Red Sea sponge *Suberea* species. In addition, five known compounds including aerothionin (**3**) [23,24,25], homoaerothionin (**4**) [2], aeroplysinin-1 (**5**) [26], aeroplysinin-2 (**6**) [27] and subereaphenol C (**7**) with a revised structure as ethyl 2-(2,4-dibromo-3,6-dihydroxyphenyl)acetate) [13] (Figure 1) were isolated from the sponge. Subereamollines C and D differ from the previously reported subereamollines A and B [13] in the terminal ester functionality at the side chain. Subereamollines C and D possess methyl ester functionality instead of the ethyl ester moiety in subereamollines A and B [13]. Recently, the total syntheses of subereamollines A and B were accomplished [28]. Optical rotations and detailed examination of the spectroscopic data including UV, 1D (^1^H and ^13^C) and 2D (COSY, multiplicity-edited HSQC and HMBC) and HRESIMS, secured the assignment of these compounds. Aerothionin (**3**) and subereaphenol C (**7**) showed strong antiproliferation activity against HeLa cell line with IC_50_ values of 29 and 13.3 µM, respectively. Additionally, aeroplysinin-2 (**6**) displayed potent antimigratory activity against the human breast cancer cell line MDA-MB-231 with IC_50_ of 18 µM.

## 2. Results and Discussion

### 2.1. Purification of Compounds **1**–**7**

The freeze-dried sponge was extracted with MeOH and the resulted extract was defatted with *n*-hexane. The defatted extract was partitioned between 60% MeOH and CH_2_Cl_2_. The CH_2_Cl_2_ extracts was subjected to partition on normal SiO_2_ VLC, size exclusion chromatography on Sephadex LH-20 and final HPLC purification on ODS RP semipreparative column to afford compounds **1**–**7** (Figure 1).

### 2.2. Structure Elucidation of Compound **1**

**Figure 2:**
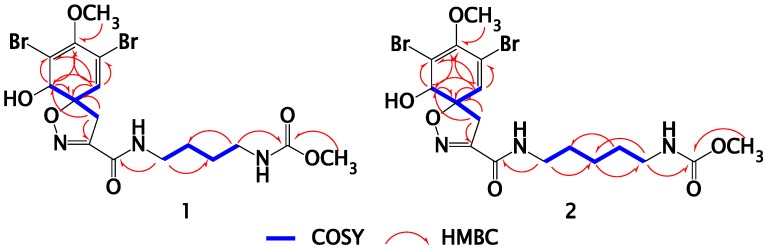
Selected COSY and HMBC correlations for **1** and **2**.

Compound **1** (Figure 1) was purified as an optically active white amorphous powder. Its ESIMS spectrum showed three ion peaks at *m/z* 531.9, 533.9, and 535.9 in the ratio of 1:2:1, respectively suggesting the di-brominated nature of the molecule. Its molecular formula was assigned as C_16_H_21_Br_2_N_3_O_6_ based on HRESIMS data (*m/z* 531.9695, [M + Na]^+^), suggesting seven degrees of unsaturation. The ^13^C-NMR spectrum of **1** (Table 1) displayed signals for 16 carbons including two carbonyls, five quaternary carbons, two methines, five methylenes, and two methyls as assigned from a multiplicity-edited HSQC experiment. Comparison of the ^1^H (Supplementary Figure S1) and ^13^C (Supplementary Figure S2) NMR data of **1** (Table 1) with those reported for subereamolline A [13] showed identical similarity of all signals with the replacement of the ethyl ester in suberemolline A with a methyl ester in **1** at δ_H_/δ_C_ 3.60/52.4 (Table 1). The assignment of all signals in **1** was unambiguously secured by extensive study of the COSY (Supplementary Figure S3), multiplicity-edited HSQC (Supplementary Figure S4) and HMBC (Supplementary Figure S5) experiments (Table 1 and Figure 2), completing the assignment of **1**. Compound **1** is reported here for the first time from a natural source and is considered as a new compound. The name subereamolline C was given to **1**.

**Table 1:** NMR data and HMBC correlations of compound **1** (CD_3_OD).

Position	δ_C_ (mult.) ^a^	δ_H_ [mult., *J* (Hz)]	HMBC (H→C#) ^b^
1	75.5 (CH)	4.07 (s)	H-5, H_2_-7
2	122.8 (qC)		H-5
3	149.3 (qC)		H-1, H-5, H_3_-16
4	114.2 (qC)		H-1, H-5
5	132.3 (CH)	6.42 (d, 0.6)	H-1, H_2_-7
6	92.3 (qC)		H-1, H-5, H_2_-7
7	40.2 (CH_2_)	3.76 (d, 18.0), 3.09 (d, 18.0)	H-1, H-5
8	155.3 (qC)		H_2_-7
9	161.5 (qC)		H_2_-10
10	40.1 (CH_2_)	3.28 (t, 6.6)	H_2_-11, H_2_-12
11	27.7 (CH_2_)	1.56 ( m)	H_2_-10, H_2_-12, H_2_-13
12	28.3 (CH_2_)	1.50 (m)	H_2_-10, H_2_-13
13	41.3 (CH_2_)	3.10 (t, 6.6)	
14	159.6 (qC)		H_2_-13, H_3_-15
15	52.4 (CH_3_)	3.60 (s)	H_2_-13
16	60.4 (CH_3_)	3.71 (s)	

^a^: Multiplicities were deduced from DEPT and multiplicity-edited HSQC; ^b^: HMBC correlations are from proton(s) stated to the indicated carbons.

### 2.3. Structure Elucidation of Compound **2**

The molecular formula of compound **2** (Figure 1) was assigned as C_17_H_23_Br_2_N_3_O_6_ from the HRESIMS pseudomolecular ion peak at *m/z* 545.9851 [M + Na]^+^. Compound **2** is 14 mass unit larger than **1**, suggesting the presence of an additional methylene unit in the **2**. Similarly, comparison of the ^1^H (Supplementary Figure S6) and ^13^C-NMR (Supplementary Figure S7) (Table 2) of **2** with those reported for subereamolline B [13] showed close similarity with the replacement of the ethyl ester moiety in subereamolline B with a methyl ester functionality in **2** at δ_H_/δ_C_ 3.60/52.4 (Table 2). In addition, the assignment of all protonated and quaternary carbons of **2** were secured from the COSY (Supplementary Figure S8), multiplicity-edited HSQC (Supplementary Figure S9) and HMBC (Supplementary Figure S10) experiments (Table 2 and Figure 2), completing the assignment of **2**. This compound is reported here for the first time from a natural source and therefore it is considered as a new natural products and it was given the generic name subereamolline D.

**Table 2:** NMR data and HMBC correlations of compound **2** (CD_3_OD).

Position	δ_C_ (mult.)	δ_H_ [mult., *J* (Hz)]	HMBC (H→C#)
1	75.5 (CH)	4.05 (s)	H-5, H_2_-7
2	122.8 (qC)		H-5
3	149.3 (qC)		H-1, H-5, H_3_-17
4	114.2 (qC)		H-1, H-5
5	132.3 (CH)	6.41 (d, 0.6)	H-1, H_2_-7
6	92.3 (qC)		H-1, H_2_-7
7	40.2 (CH_2_)	3.75 (d, 18.0), 3.09 (d, 18.0)	H-1, H-5
8	155.3 (qC)		H_2_-7
9	161.5 (qC)		H_2_-10
10	40.3 (CH_2_)	3.26 (t, 7.2)	H_2_-11, H_2_-12
11	30.0 (CH_2_)	1.56 (quin, 7.2)	H_2_-10
12	25.0 (CH_2_)	1.34 (m)	H_2_-10, H_2_-13, H_2_-14
13	30.5 (CH_2_)	1.49 (quin, 7.2)	H_2_-10, H_2_-14
14	41.6 (CH_2_)	3.07 (t, 7.2)	
15	159.6 (qC)		H_2_-14, H_3_-16
16	52.4 (CH_3_)	3.60 (s)	
17	60.4 (CH_3_)	3.71 (s)	

^a^: Multiplicities were deduced from DEPT and multiplicity-edited HSQC; ^b^: HMBC correlations are from proton(s) stated to the indicated carbons.

### 2.4. Structure Elucidation of Compounds **3**–**7**

The known compounds **3**–**7** (Figure 1) were identified as aerothionin (**3**) [23,24,25], homoaerothionin (**4**) [2], aeroplysinin-1 (**5**) [26], aeroplysinin-2 (**6**) [27] and subereaphenol C [13]. The structures of all compounds were established by extensive study of their spectral data including 1D and 2D NMR as well as high-resolution mass spectroscopy. However, the structure of **7** was revised and assigned as ethyl 2-(2,4-dibromo-3,6-dihydroxyphenyl)acetate based on reinvestigation of its NMR data and the revised structure was shown in Figure 1.

### 2.5. Biological Activities of the Isolated Compounds

All compounds were found to be >98% pure based on HPLC purity check. Compounds **1**–**7** were evaluated for their antimigratory activity against the highly metastatic triple negative human breast cancer cells MDA-MB-231 and their antiproliferation activity against Hela cell line. In the wound healing assay to evaluate the migration of highly metastatic triple negative human breast cancer cells MDA-MB-231, compound **6** showed significant antimigratory activity with IC_50_ value of 18 μM compared to 43.4 μM showed by the positive control *Z*-4-ethylthio-phenylmethylene hydantoin (*S*-Ethyl) (Table 3). All other compounds were weakly active against this cell line. These results clearly suggest that compound **6** could be a potential hit for future development of drugs to control metastatic breast cancer. On the other hand, in the antiproliferative assay, aerothionin (**3**) and subereaphenol C (**7**) showed potent antiproliferation activity against HeLa cell line with IC_50_ values of 29 and 13.3 µM, respectively. The results of the antimigratory and antiproliferation activities of **1**–**7** were shown in Table 3. Surprisingly, subereamolline C, the methyl ester congener of the potent antimigratory subereamoline A [13] was weakly active compared to subereamolline A [13].

**Table 3:** Antimigatory and antiproliferative activities of **1**–**7**.

Compound	IC_50_ (μM)	
	Antimigratory Activity (MDA-MB-231)	Antiproliferative Activity (HeLa Cells)
**1**	>50	>50
**2**	>50	>50
**3**	>50	29
**4**	NT	NT
**5**	NT	NT
**6**	18.0	>50
**7**	>50	13.3
*S*-Ethyl *	43.4	NT
Paclitaxel *	NT	0.0017

*: positive controls; NT = Not tested.

## 3. Experimental Section

### 3.1. General Experimental Procedures

Optical rotation was measured on a JASCO DIP-370 digital polarimeter (Jasco Co., Tokyo, Japan) at 25 °C at the sodium D line (589 nm). UV spectrum was recorded on a Hitachi 300 spectrometer (Hitachi High-Technologies Corporation, Kyoto, Japan). NMR spectra were determined on BRUKER Unity INOVA 600 instruments (600 MHz for ^1^H and 150 MHz for ^13^C-NMR) (Bruker BioSpin, Billerica, MA, USA). NMR chemical shifts are expressed in parts per million (ppm) referenced to CD_3_OD solvent signals (δ 3.29 for ^1^H and δ 49.0 for ^13^C). Positive ion ESIMS mass spectral data were obtained with a Micromass Q-tof equipped with lockspray mass spectrometer using Leucine Enkaphalin at *m/z* 556.2771 [M + H]^+^ as a reference mass. The HPLC separation and quantitation were made on a RP18, 250 × 10 mm, 5 µm Cosmosil ARII column. Precoated silica gel G-25 UV_254_ plates were used for thin layer chromatography and silica gel 60, 230–40 μm mesh (E. Merck, Darmstadt, Germany) and Sephadex LH-20 (Pharmacia, Piscataway, NJ, USA) were used for column chromatography.

### 3.2. Biological Materials

The marine sponge *Suberea* species was collected off Yanbu at the Saudi Red Sea at depths between 15 and 28 m (N024°13′49.1″ E037°42′96.4″) on May 2013. The sponge forms encrusting mass of 5–7 cm with conulose surface. The conules were low but sharp due to projecting strong fibers, about 8–10 mm apart. The oscules are large, approximately 1.0 cm in diameter, positioned at the summit of the fragment. In life, the sponge is yellowish green in color with a yellowish interior. In preserved condition, the sponge turns completely into black. The interior of the sponge is cavernous. The ectosomal region is a distinctly denser mass of collagen and crowded large spherulous cells, whereas deeper in the body the organic parts are only lightly collagenous and they are charged with many small calcareous nodules. The skeleton consists of thick pitched fibers, which run for long distances without branching or anastomosing. The fibers measure approximately 400 μm in diameter, of which the pith occupies 75%. The bark consists of several thick laminae of amber colored spongin. This sponge conforms in most aspects (shape, surface characters and fibers) to the description of the type of *Suberea* sp. (Row), 1911 (as *Aplysina*) (class Demospongiae, order Verongida, family Aplysinellidae). A fragment is kept in the collections of the Naturalis Biodiversity Center at Leiden, The Netherlands under the registration number RMNHPOR 9183. Another voucher specimen was deposited in the Red Sea Invertebrates Collection of the Department of Natural Products, Faculty of Pharmacy at King Abdulaziz University under the code number DY-KSA-32.

### 3.3. Purification of Compounds **1**–**7**

The lyophilized sponge material (540 g) was extracted with MeOH (3 × 1500 mL) and the resulted extracts were evaporated under reduced pressure. The crude extract was dissolved in 90% MeOH and extracted with *n*-hexane. The resulting methanolic-aqueous layer was diluted with H_2_O to 60% MeOH followed by extraction with CH_2_Cl_2_ which upon evaporation yielded a brown residue (7.5 g). The CH_2_Cl_2_ extract was fractionated by VLC on Silica gel column using *n*-hexane/CH_2_Cl_2_/MeOH gradients to afford five main subfractions (A–E). Fraction C (1.3 g) was subjected to partition on a Sephadex LH-20 column using MeOH to afford three subfractions (C1–C3). Fraction C-3 (210 mg) was partitioned again on a Sephadex LH-20 column using MeOH and the main fraction (75 mg) was purified on ODS HPLC column (RP18, 5 μm, ARII Cosmosil, 250 × 10 mm, Waters) using 50% CH_3_CN in H_2_O to afford compounds **1** (7.5 mg), **2** (9.4 mg), **5** (13 mg) and **7** (9 mg). Similarly, fraction C1 (250 mg) was partitioned on a Sepahdex LH-20 column using MeOH and the resulting main fraction (85 mg) was purified on ODS HPLC column (RP18, 5 μm, ARII Cosmosil, 250 × 10 mm) using 45% CH_3_CN in H_2_O to afford compounds **3** (40 mg), **4** (12 mg) and **6** (7 mg).

### 3.4. Biological Evaluation of the Compounds

#### 3.4.1. Evaluation of the Antimigratory of **1**–**7** Using Wound Healing Assay

The wound healing assay is a simple method for evaluating directional cell migration *in vitro*. All compounds were tested for ability to inhibit the migration of highly metastatic triple negative human breast cancer cells MDA-MB-231 using wound-healing assay model. A vehicle (DMSO) and *Z*-4-ethylthio-phenylmethylene hydantoin (*S*-Ethyl) were used as negative and positive controls. The assay was conducted as described previously [29]. Briefly, cells were plated on sterile 24-well plates and allowed to form a confluent monolayer per well (>90% confluence) overnight. Wounds were then inflicted in each cell monolayer using a sterile 200 μL pipette tip. The media was removed and cells were washed twice with PBS and once with fresh RPMI medium. Test compounds at the desired concentrations were prepared in fresh medium (0.0% or 0.5% FBS) and were added to wells in triplicate. The incubation was carried out for 24 h, after which the medium was removed and cells were washed, fixed and stained using Diff-Quick™ staining (Dade Behring Diagnostics, Aguada, Puerto Rico). Cells which migrated across the inflicted wound were counted under the microscope in at least five randomly selected fields (magnification: 400×). The results were shown in Table 3.

#### 3.4.2. Evaluation of Antiproliferaive and Cytotoxic Activities against HeLa Cells

The effects of the compounds **1**–**7** on HeLa cell proliferation and cytotoxicity were evaluated using the sulforhodamine B (SRB) assay [30,31,32]. HeLa cells were grown in Basal Medium Eagle (BME) containing Earle’s salts, 10% FBS and 50 μg/mL gentamycin sulfate. Cells were plated at a density of 2500 cells per well in a 96-well plate and allowed to adhere and grow for 24 h before compounds were added. The compounds were solubilized in DMSO and added to a final DMSO concentration of 1% in both test wells and vehicle controls. The cells were incubated with compounds or vehicle for an additional 48 h. The IC_50_, the concentrations required to cause a 50% inhibition of cell proliferation, was calculated from the log dose response curves. The values represent the average of 3–4 independent experiments, each conducted in triplicate ± SEM. Cytotoxicity was determined by a cell density lower than that measured at the time of drug addition. Paclitaxel was used as a positive control.

**Subereamolline C (1)**: White amorphous powder; [α]_D_ +150 (*c* 0.7, MeOH); UV (MeOH) λ_max_ nm (log ε) 280 (3.60), 230 (3.70), 207 (3.70); ^1^H and ^13^C-NMR data, see Table 1; positive HRESIMS *m/z* 531.9695 (calcd for C_16_H_21_^79^Br_2_N_3_NaO_6_, [M + Na]^+^, 531.9694).

**Subereamolline D (2)**: White amorphous powder; [α]_D_ +136 (*c* 0.5, MeOH); UV (MeOH) λ_max_ nm (log ε) 280 (3.60), 234 (3.75), 220 (3.80); ^1^H and ^13^C-NMR data, see Table 1; positive HRESIMS *m/z* 545.9851 (calcd for C_17_H_23_^79^Br_2_N_3_NaO_6_, [M + Na]^+^, 545.9851).

## 4. Conclusions

Investigation of the organic extract of the Red Sea marine Verongid sponge *Suberea* species afforded two new dibrominated alkaloids, subereamollines C (**1**) and D (**2**), together with the known compounds aerothionin (**3**), homoaerothionin (**4**), aeroplysinin-1 (**5**), aeroplysinin-2 (**6**) and subereaphenol C (**7**) with a revised structure as ethyl 2-(2,4-dibromo-3,6-dihydroxyphenyl)acetate. The structure determinations of the compounds were established by detailed examination of their spectroscopic data including UV, 1D (^1^H and ^13^C), 2D (COSY, multiplicity-edited HSQC and HMBC) NMR and HRESIMS. Aerothionin (**3**) and subereaphenol C (**7**) showed potent antiproliferation activity against HeLa cell line with IC_50_ values of 29 and 13.3 μM, respectively. Aeroplysinin-2 (**6**) displayed potent antimigratory activity against the human breast cancer cell line MDA-MB-231 with IC_50_ of 18 μM.

## Supplementary Files

Supplementary File 1
