# Supplementary material for: Bioactive Secondary Metabolites from the Red Sea Marine Verongid Sponge Suberea Species
Source: Mar Drugs. 2015 Mar 24;13(4):1621–31. doi: 10.3390/md13041621 (PMC4413177; doi:10.3390/md13041621)
Supplement: Supplementary File 1 [file marinedrugs-13-01621-s001.pdf]

Supplementary Information

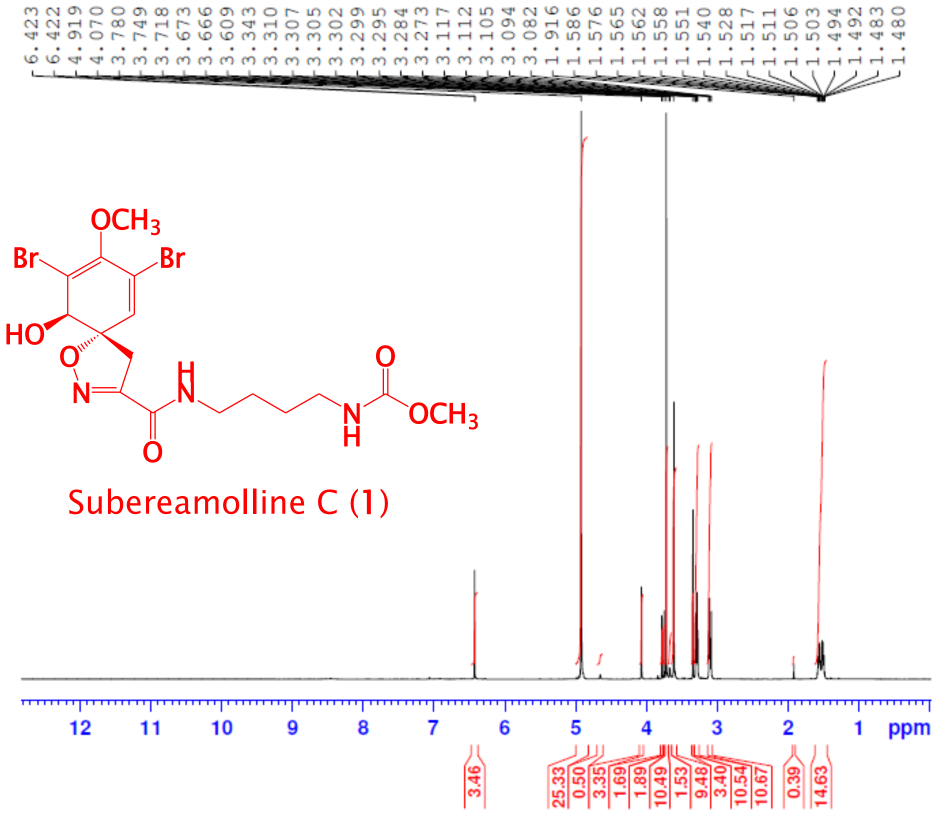

Figure S1. ^{1}H-NMR spectrum of compound **1** (CD3OD).

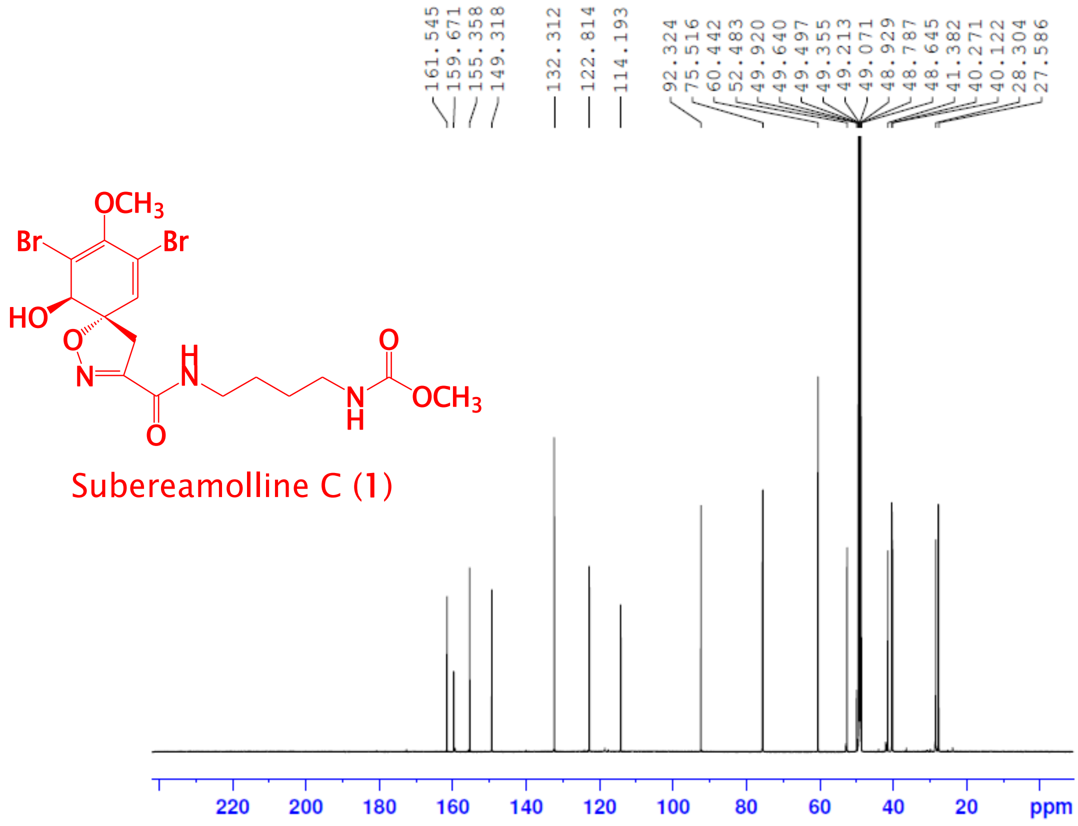

Figure S2. ^{13}C-NMR spectrum of compound **1** (CD3OD).

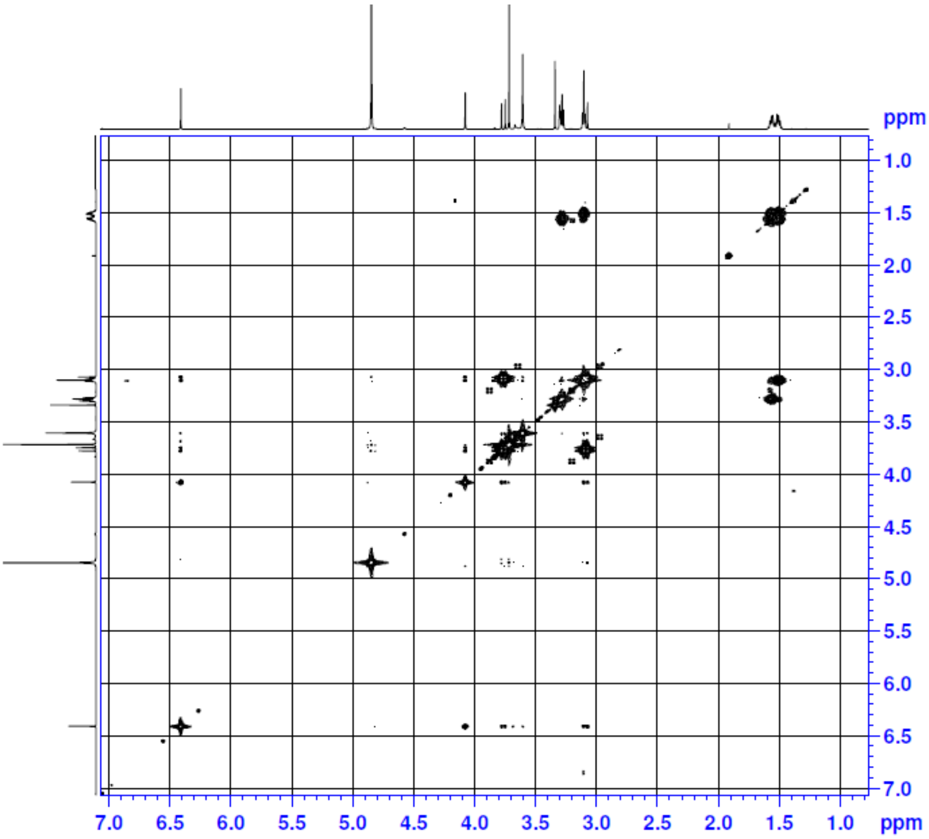

Figure S3. ^{1}H-^{1}H COSY spectrum of compound **1** (CD_3OD).

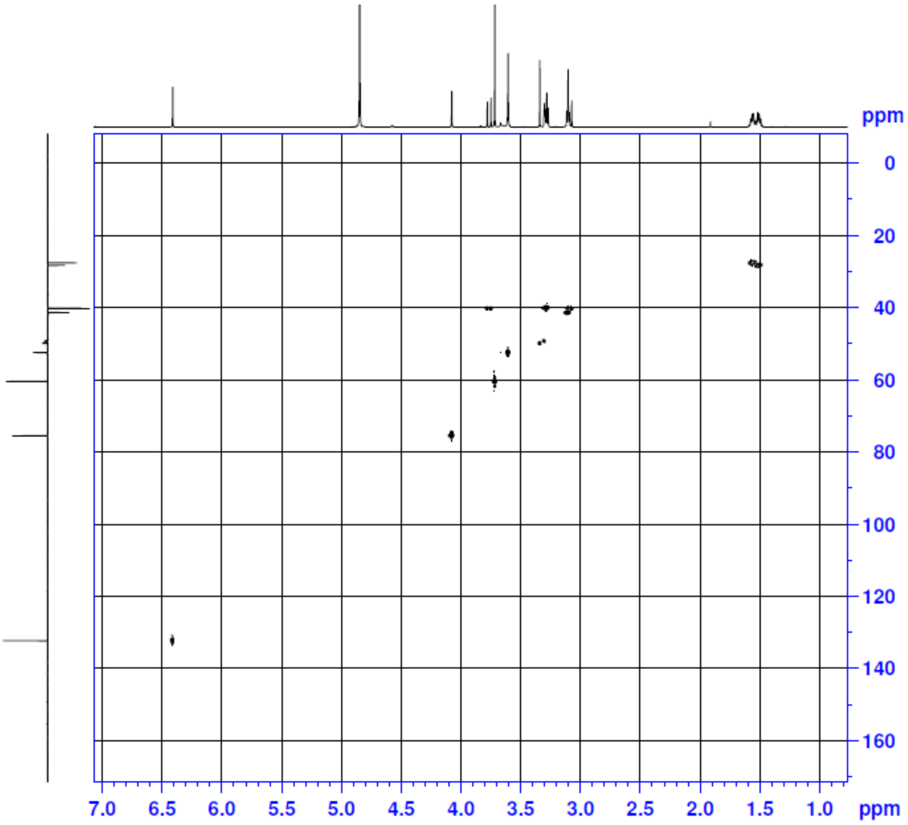

Figure S4. Multiplicity-edited HSQC spectrum of compound **1** (CD_3OD).

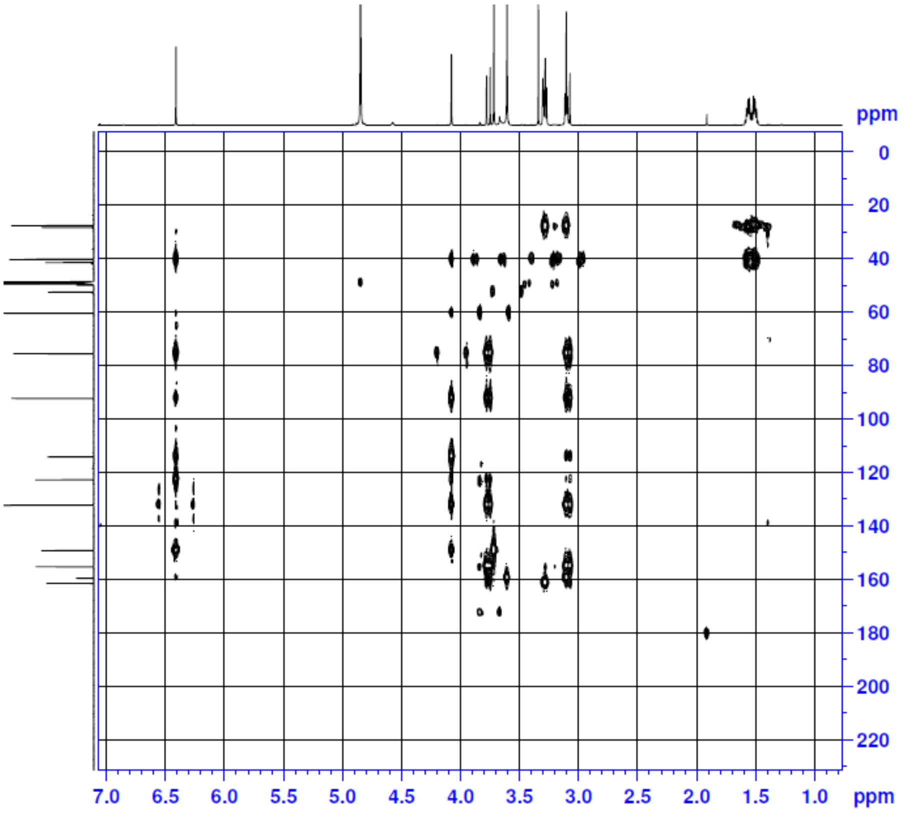

Figure S5. HMBC spectrum of compound **1** (CD_3OD).

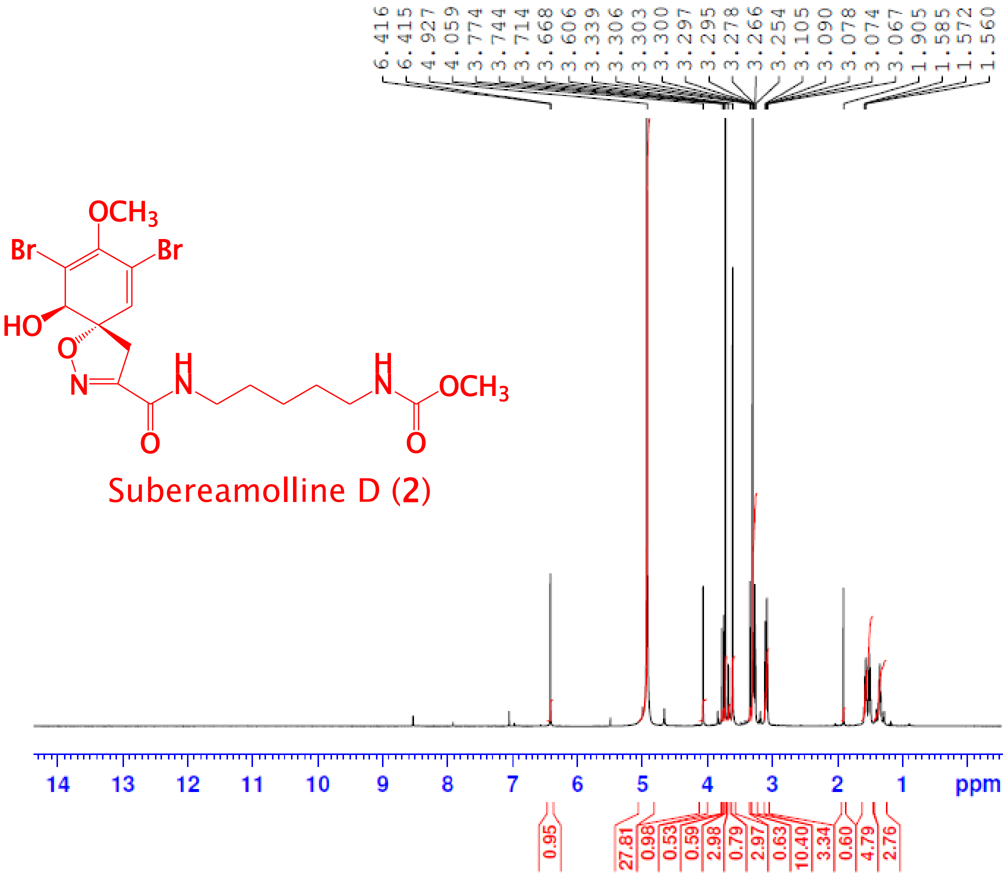

Figure S6. ^{1}H-NMR spectrum of compound **2** (CD_3OD).

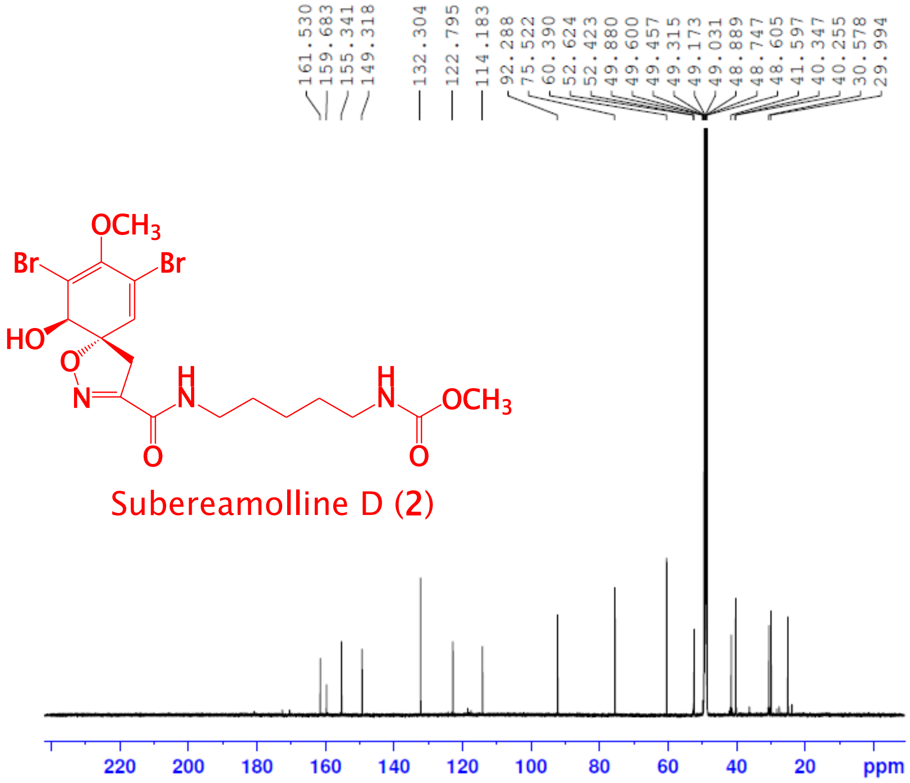

Figure S7. ^{13}C-NMR spectrum of compound **2** (CD_3OD).

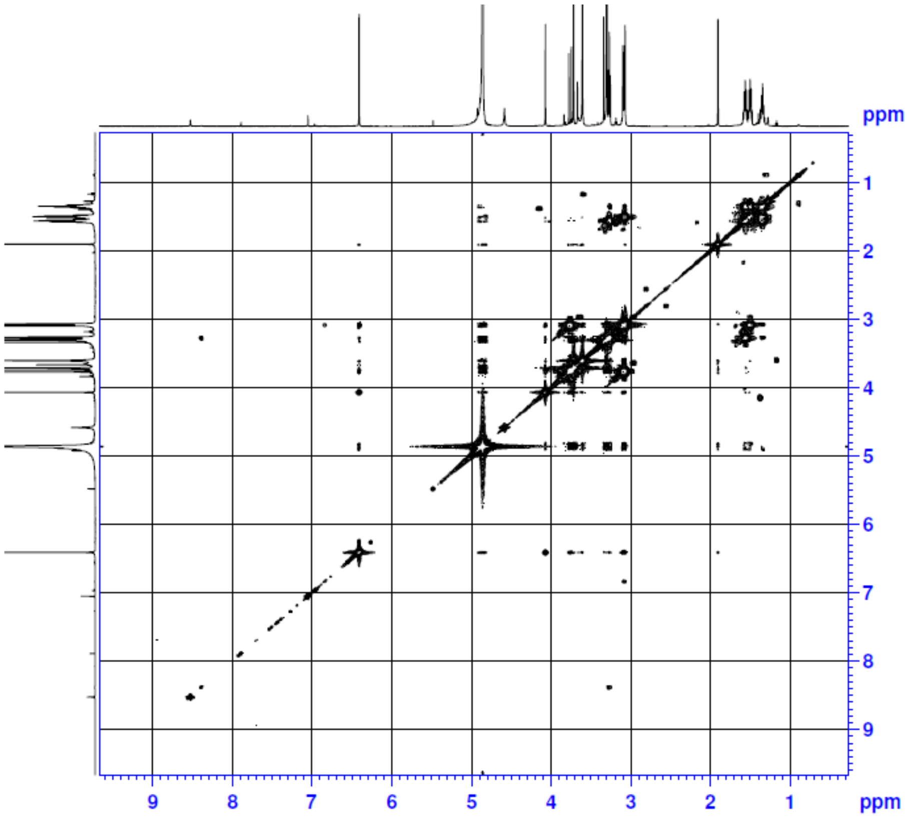

Figure S8. ^{1}H-^{1}H COSY spectrum of compound **2** (CD_3OD).

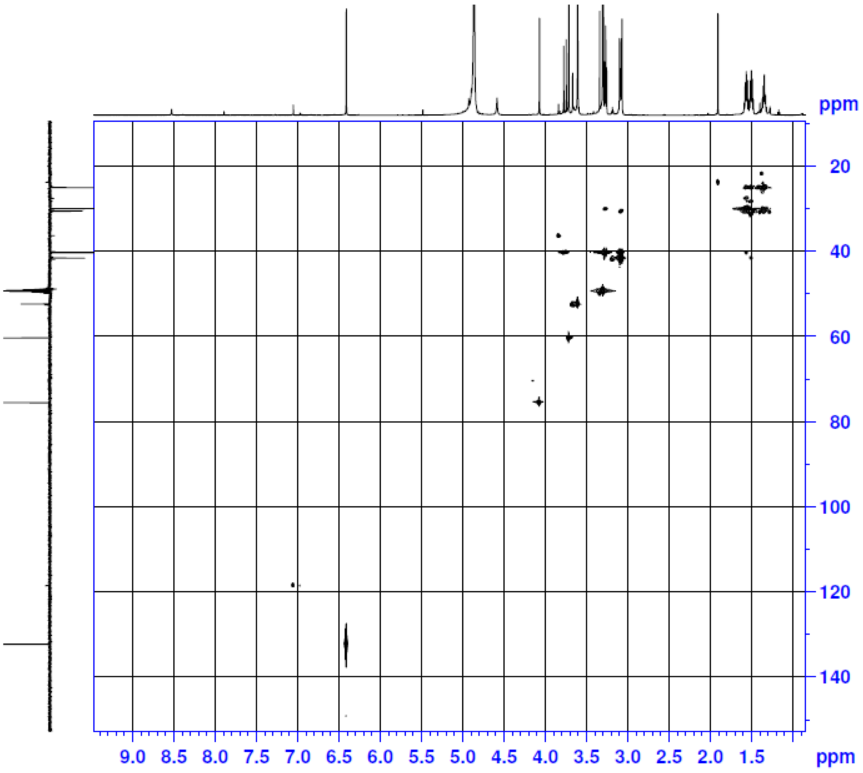

Figure S9. Multiplicity-edited HSQC spectrum of compound **2** (CD_3OD).

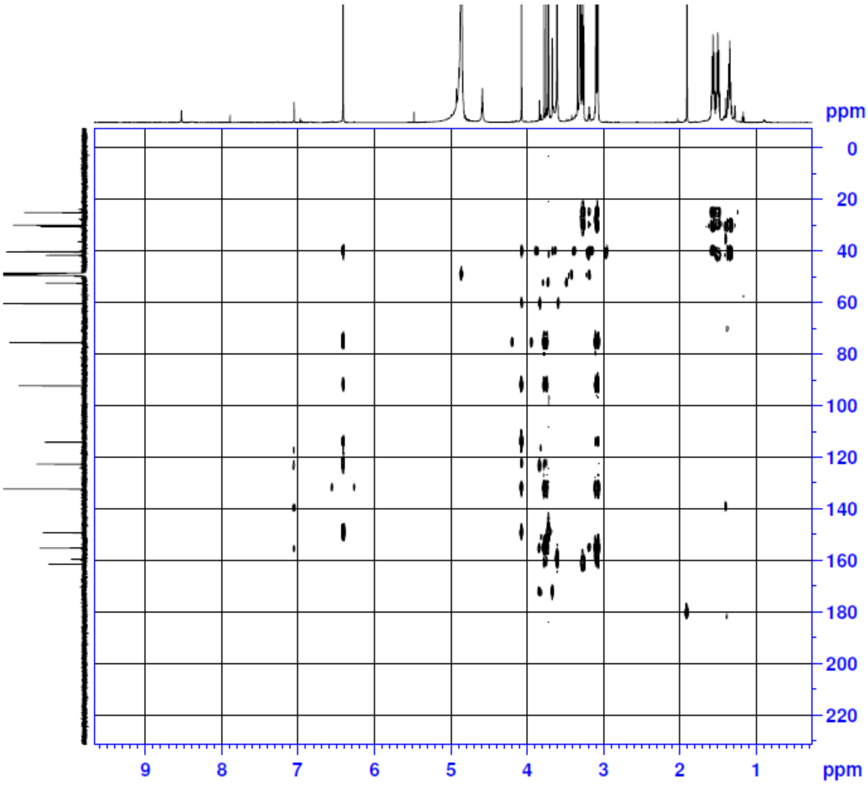

Figure S10. HMBC spectrum of compound **2** (CD_3OD).

© 2015 by the authors; licensee MDPI, Basel, Switzerland. This article is an open access article distributed under the terms and conditions of the Creative Commons Attribution license (http://creativecommons.org/licenses/by/4.0/).
